# Perioperative Bronchospasm Mimicking Anaphylaxis in a Patient With Undiagnosed Asthma Requiring Venovenous ECMO Support

**DOI:** 10.1155/crcc/5583594

**Published:** 2026-11-10

**Authors:** Majken Christensen, Birgitte Louise Bech Melchiors, Lene Heise Garvey, Niels Secher

**Affiliations:** ^1^ Department of Anaesthesia Operation and Intensive Care Regional Hospital Horsens Horsens Region Midtjylland Denmark; ^2^ Danish Anaesthesia Allergy Centre Allergy Clinic Copenhagen University Hospital Gentofte Copenhagen Denmark gentoftehospital.dk

**Keywords:** anaphylaxis differential diagnosis, case report, perioperative bronchospasm, undiagnosed asthma, venovenous extracorporeal membrane oxygenation

## Abstract

Severe perioperative bronchospasm is rare but may mimic anaphylaxis and lead to life‐threatening respiratory failure. We report a 38‐year‐old woman with obesity and a history of active smoking who developed refractory bronchospasm immediately after induction of general anaesthesia for surgical debridement. Despite intensive treatment with bronchodilators, corticosteroids, magnesium, ketamine and adrenaline, ventilation remained critically impaired with severe hypercapnic respiratory acidosis. Venovenous extracorporeal membrane oxygenation (VV‐ECMO) was initiated because of persistent respiratory failure. Serum tryptase levels were normal, and no clinical signs of anaphylaxis were observed. Subsequent pulmonary function testing demonstrated severe reversible airflow obstruction, leading to a diagnosis of previously unrecognised asthma. This case highlights that undiagnosed asthma may present as catastrophic perioperative bronchospasm and that timely consideration of VV‐ECMO may be lifesaving in refractory cases.

## 1. Introduction

Bronchospasm during general anaesthesia is a well‐known and potentially life‐threatening complication, characterised by prolonged expiration, wheezing and elevated peak airway pressures. If untreated, it may result in hypoxaemia, hypotension and increased perioperative morbidity and mortality [1].

The reported incidence of perioperative bronchospasm during general anaesthesia is approximately 0.2% in large epidemiological series [2]. In patients with asthma, a population‐based cohort reported a frequency of 1.7%, with higher rates observed in those with active disease [3].

Risk factors include poorly controlled or active asthma, COPD and active smoking. Intraoperative triggers include airway instrumentation, inadequate depth of anaesthesia, airway secretions or obstruction, aspiration and the administration of histamine‐releasing or bronchoconstrictive agents [1].

Perioperative anaphylaxis is a rare but important differential diagnosis in severe or treatment‐resistant bronchospasm, and prompt recognition is crucial [4].

We describe a case of a patient without previously known pulmonary disease who developed severe bronchospasm during general anaesthesia, requiring extracorporeal membrane oxygenation (ECMO).

## 2. Case Report

A 38‐year‐old woman with obesity (BMI 38), active smoking and sertraline‐treated anxiety was scheduled for her third general anaesthesia for surgical debridement of an infected haematoma of the right buttock.

The first procedure was uneventful except for elevated peak airway pressures (31 cm H₂O). During the second procedure, bronchospasm occurred at induction, followed by desaturation during recovery.

For the third procedure, anaesthesia was induced with remifentanil 100 *μ*g, propofol 200 mg and succinylcholine 100 mg. Shortly after induction, severe bronchospasm developed with rising airway pressures. Suspected endotracheal tube malposition prompted reintubation without improvement. Anaesthesia was converted to sevoflurane, and repeated inhalations of salbutamol and the combination of ipratropium bromide and fenoterol were administered.

Shortly after induction, the patient became briefly hypotensive (80/50 mmHg), refractory to intravenous ephedrine 10 mg, necessitating initiation of a phenylephrine infusion (0.1 mg/mL at 20 mL/h). However, no persistent cardiovascular collapse, cutaneous manifestations or other clinical signs of anaphylaxis were observed.

During attempted emergence from anaesthesia, bronchospasm worsened with high peak airway pressures and low tidal volumes. Treatment was escalated with inhaled adrenaline 1 mg, intravenous magnesium sulphate 10 mmol, ketamine 50 mg, continuous salbutamol infusion, theophylline 240 mg, rocuronium 20 mg, clemastine 2 mg and methylprednisolone 500 mg in divided doses. An adrenaline infusion was subsequently initiated at 0.2 *μ*g/kg/min. Despite maximal therapy, the patient remained critically ill, with tidal volumes decreasing to approximately 200 mL and peak airway pressure increasing from 27 to 40 cm H₂O. The first available arterial blood gas showed respiratory acidosis with pH 7.18 and PaCO₂ 8.7 kPa. Respiratory acidosis subsequently worsened, with a lowest recorded pH of 6.97 and a corresponding PaCO₂ of 14.4 kPa.

As severe hypercapnic respiratory acidosis and critically impaired ventilation persisted despite extensive treatment, VV‐ECMO was initiated at 17:00, approximately 5 h and 20 min after the onset of bronchospasm. By 19:56, pH had normalised to 7.37 and PaCO₂ had decreased to 4.5 kPa. Detailed physiological data and interventions are presented in Table S2.

Serum tryptase measured 2 h after the event was 6.09 *μ*g/L, whereas the baseline level measured 3 days later was 6.87 *μ*g/L; both were within the normal reference range.

Subsequent pulmonary function testing demonstrated FEV₁ 39% of predicted and FEV₁/FVC 46%. Following inhaled salbutamol, FEV₁ increased to 57% and FEV₁/FVC to 58%, corresponding to an absolute improvement of 520 mL (47.7%). A diagnosis of previously unrecognised asthma was established, and appropriate inhalation therapy was initiated.

The patient was referred to the Danish Anaesthesia Allergy Centre, the Danish national reference centre for investigation of perioperative allergic reactions, comprising in vitro testing, skin testing and ultimately drug provocation of all suspected drugs administered prior to the reaction [5]. Allergy investigations for cloxacillin, propofol, remifentanil, ephedrine, lidocaine gel 2%, chlorhexidine, latex, ethylene oxide and excipients were all negative. Skin testing and basophil activation testing for succinylcholine and rocuronium were negative, with a high negative predictive value [6]. However, as asthma control was insufficient at the time of evaluation and given the severity of the perioperative reaction requiring ECMO, drug provocation testing with succinylcholine and rocuronium was deemed unsafe. IgE‐mediated allergy to neuromuscular blocking agents was therefore considered unlikely but could not be definitively excluded in the absence of drug provocation testing. Future use of neuromuscular blocking agents should therefore be undertaken with caution in a controlled setting.

## 3. Discussion

This case illustrates how previously unrecognised asthma may present as severe, life‐threatening bronchospasm during general anaesthesia. In retrospect, active smoking, obesity and elevated airway pressures during the first anaesthetic were potential warning signs of underlying airway hyperreactivity [1]. The bronchospasm during the second anaesthetic should therefore have prompted pulmonary evaluation, including spirometry where clinically feasible, potentially enabling earlier diagnosis and optimisation of the underlying asthma.

This case underscores the importance of reviewing previous anaesthetic records and specifically assessing prior airway reactions, chronic cough, wheezing, nocturnal symptoms and other unexplained respiratory events [1]. Such events should be clearly documented, communicated and systematically followed up before subsequent procedures.

Despite intensive pharmacological and ventilatory treatment, the patient remained critically bronchospastic, with a peak airway pressure of 40 cm H₂O and calculated tidal volumes of approximately 180–260 mL. Attempts to maintain minute ventilation failed to prevent progressive CO₂ retention, with PaCO₂ reaching 14.4 kPa and pH decreasing to 6.97. Although oxygenation was also impaired, the principal indication for VV‐ECMO was refractory hypercapnic respiratory failure rather than hypoxaemia.

ELSO guidance recommends considering VV‐ECMO in severe, potentially reversible hypercapnic respiratory failure with a pH below 7.25 despite optimal conventional management [7]. The recognised risks of VV‐ECMO, particularly bleeding and cannulation‐related complications, were balanced against the immediate risks of profound acidaemia, further ventilator‐induced lung injury and cardiovascular deterioration. Because the airway obstruction was considered reversible and no major contraindication to extracorporeal support was present, the anticipated benefit was considered to outweigh these risks. The rapid decrease in PaCO₂ following VV‐ECMO initiation illustrates its role as temporary respiratory support while the airway obstruction resolved.

Published evidence on ECMO for near‐fatal asthma is derived largely from reports of nonperioperative status asthmaticus and registry analyses that do not specifically address perioperative presentations [8, 9]. A previously reported case of undiagnosed asthma presenting with bronchospasm during anaesthetic induction was successfully managed pharmacologically without extracorporeal support [10]. In contrast, Chang described refractory status asthmaticus immediately following tracheal intubation that required VV‐ECMO, although this occurred in a patient with pre‐existing asthma in whom anaphylaxis was not initially suspected [11]. The present case extends this literature by describing previously unrecognised asthma presenting as catastrophic peri‐induction bronchospasm that mimicked anaphylaxis and ultimately required emergency VV‐ECMO (Table S1).

Perioperative anaphylaxis was considered an important differential diagnosis. However, the absence of cutaneous manifestations or persistent cardiovascular collapse, together with normal acute and baseline serum tryptase levels, argued against an IgE‐mediated mechanism. Although normal tryptase levels do not completely exclude perioperative anaphylaxis, they reduce the likelihood of an IgE‐mediated reaction [4].

Also, subsequent allergy evaluation did not confirm any allergy, although final provocation testing with NMBAs was not performed due to the patient′s high‐risk profile. An NMBA‐related reaction could therefore not be definitively excluded. Isolated bronchospasm during anaesthesia is often non‐allergic in origin, either due to mechanical airway manipulation or the pharmacological effects of various histamine‐releasing drugs in a hyperresponsive airway [12, 13]. Non‐allergic differential diagnoses for isolated bronchospasm without an increase in tryptase include inadequate depth of anaesthesia, tracheal tube malposition or aspiration, undiagnosed or uncontrolled asthma/COPD and hyperreactive airways associated with predisposing factors such as asthma, smoking or viral infection [14].

For future anaesthesia, the patient should therefore be regarded as high risk until adequate asthma control has been achieved. If anaesthesia is necessary, it should be undertaken with extreme caution in a controlled setting with immediate access to advanced airway management and extracorporeal support. Elective procedures should be postponed until asthma is well controlled, and anaesthetic planning should aim to minimise airway manipulation and bronchial stimulation. Caution with the administration of NMBAs is currently recommended, and alternative anaesthetic techniques should be considered where feasible.

In conclusion, this case demonstrates that previously unrecognised asthma may present as catastrophic perioperative bronchospasm and mimic anaphylaxis. Careful review of previous anaesthetic records and preoperative assessment of suspected airway disease may enable earlier diagnosis and optimisation. In refractory hypercapnic respiratory failure, timely initiation of VV‐ECMO may provide lifesaving temporary support while the bronchospasm resolves.

## Funding

No funding was received for this manuscript.

## Consent

Written informed consent was obtained from the patient for publication of this case report and the accompanying clinical data.

## Conflicts of Interest

The authors have nothing to report.

## Supporting information


**Supporting Information** Additional supporting information can be found online in the Supporting Information section. Table S1: Published cases of severe bronchospasm or status asthmaticus during anaesthesia. Table S2: Timeline of physiological parameters, ventilatory settings, arterial blood gas results and therapeutic interventions.

## Data Availability

The data that support the findings of this study are available on request from the corresponding author. The data are not publicly available due to privacy or ethical restrictions.
