# Supplementary material for: Perioperative Bronchospasm Mimicking Anaphylaxis in a Patient With Undiagnosed Asthma Requiring Venovenous ECMO Support
Source: Case Rep Crit Care. 2026 Nov 10;2026:5583594. doi: 10.1155/crcc/5583594 (PMC13752561; doi:10.1155/crcc/5583594)
Supplement: Supplementary file 1 [file CRCC-2026-5583594-s001.docx]

**Supplementary Table 1. Comparison with selected reports of perioperative bronchospasm and ECMO-supported near-fatal asthma**

| **Study** | **Trigger** | **Asthma status** | **Management** | **Outcome** |
| --- | --- | --- | --- | --- |
| **Current case**  **Case report** | Catastrophic bronchospasm immediately following induction of general anaesthesia; perioperative anaphylaxis initially suspected | Previously unrecognised asthma, subsequently confirmed by pulmonary function testing | Inhaled and intravenous bronchodilators, corticosteroids, sevoflurane, magnesium, ketamine and adrenaline, followed by emergency VV-ECMO | Successfully weaned from VV-ECMO and subsequently diagnosed with asthma |
| **Chang, 2018** [1]  Case report | Refractory status asthmaticus immediately after tracheal intubation for thoracic endovascular aortic repair | Pre-existing asthma | Bronchodilators, corticosteroids and ventilatory support, followed by VV-ECLS | Surgery completed; VV-ECLS discontinued the following day |
| **Kim and Choi, 2020** [2]  Case report | Bronchospasm immediately after anaesthetic induction in a 10-year-old child; severe hypotension raised concern for anaphylaxis | Previously undiagnosed asthma; history of allergic rhinitis | Nebulised salbutamol, intravenous dexamethasone and adrenaline; surgery cancelled | Recovered without complications; asthma diagnosed two weeks later; ECMO not required |
| **Maqsood and Patel, 2018** [3]  Case report | Near-fatal asthma refractory to mechanical ventilation, with peak airway pressure of 55 cmH₂O and intrinsic PEEP of 14 cmH₂O | Known severe asthma | Bronchodilators, corticosteroids, intravenous salbutamol, sedation, neuromuscular blockade and sevoflurane, followed by VV-ECMO | PaCO₂ normalised within 48 hours; decannulated from VV-ECMO on day 2 |
| **Yeo et al., 2017** [4]  Retrospective observational cohort study | ELSO registry analysis of 272 adults receiving ECMO for life-threatening asthma refractory to mechanical ventilation | Asthma diagnosis recorded; perioperative context not specified | Predominantly VV-ECMO (93.9%) | 86.7% successfully weaned from ECMO; 83.5% survived to hospital discharge |

1. Chang WH. Early administration of venovenous extracorporeal life support for status asthmaticus during anaesthetic induction: case report and literature review. Open Med (Wars). 2018;13:17–21. doi:10.1515/med-2018-0003.
2. Kim BS, Choi JC. Bronchospasm during anesthesia induction in a patient with undiagnosed asthma. Asian J Pharm Nurs Med Sci. 2020;8(6). doi:10.24203/ajpnms.v8i6.6417.
3. Maqsood U, Patel N. Extracorporeal membrane oxygenation for near-fatal asthma refractory to conventional ventilation. BMJ Case Rep. 2018;2018:bcr-2017-223276. doi:10.1136/bcr-2017-223276.
4. Yeo HJ, Kim D, Jeon D, et al. Extracorporeal membrane oxygenation for life-threatening asthma refractory to mechanical ventilation: analysis of the Extracorporeal Life Support Organization registry. Crit Care. 2017;21:297. doi:10.1186/s13054-017-1886-8.

**Supplementary Table 2A. Perioperative timeline, interventions, and physiological variables**

| **Time** | **Clinical event/intervention** | **BP (mmHg)** | **HR (beats/min)** | **FiO₂** | **Ppeak/PEEP (cmH₂O)** | **EtCO₂ (kPa)** | **MV/RR** | **Calculated TV (mL)** | **Displayed MAC** |
| --- | --- | --- | --- | --- | --- | --- | --- | --- | --- |
| 11:15 | Pre-induction | 115/50 | 80 | — | — | — | — | — | — |
| 11:30 | Induction: remifentanil 100 µg IV, propofol 200 mg IV and succinylcholine 100 mg IV. Ephedrine 10 mg IV × 2 administered after induction | 70/40 | 70 | 1.00 | 27/4 | 4.3 | 7.5/10 | 750 | — |
| 11:40 | Bronchospasm documented. Cloxacillin 1 g IV administered. Inhaled salbutamol × 2 and ipratropium/fenoterol × 2 | 85/60 | 70 | — | — | — | — | — | — |
| 11:45 | Changed from TIVA to sevoflurane for bronchodilation.  Phenylephrine infusion initiated at 0,1 mg/ml 20 ml/h | 85/60 | 70 | 0.50 | 27/4 | 4.3 | 7.5/10 | 750 | 1.8 |
| 12:00 | Dexamethasone 4 mg IV, ondansetron 4 mg IV, morphine 5 mg IV and paracetamol 1 g IV | 110/65 | 60 | 0.50 | 27/4 | 4.3 | 5.8/10 | 580 | 1.6 |
| 12:15 | Phenylephrine reduced to 15 mL/h | 115/70 | 60 | 0.50 | 27/4 | 4.8 | 4.6/20 | 230 | 1.1 |
| 12:30 | Magnesium sulfate 10 mmol IV, ketamine 50 mg IV, nebulised adrenaline 1 mg and inhaled salbutamol × 2. Phenylephrine reduced to 10 mL/h | 105/75 | 70 | 0.50 | 40/3 | 6.2 | 4.6/20 | 230 | 1.1 |
| 12:45 | Persistent bronchospasm | 80/50 | 70 | 0.60 | 40/3 | 6.5 | 6.3/23 | 274 | 3.3 |
| 13:00 | Salbutamol 0.25 mg IV bolus followed by infusion at 250 µg/h | 100/50 | 75 | 0.60 | 32/3 | 6.5 | 6.7/22 | 305 | 3.3 |
| 13:15 | Theophylline 240 mg IV over 20 min and rocuronium 20 mg IV. | 95/45 | 85 | 0.60 | 35/4 | 6.6 | 5.5/20 | 275 | 3.3 |
| 13:30 | Adrenaline infusion initiated at 0.2 µg/kg/min | 105/45 | 90 | 0.80 | 35/4 | 5.6 | 6.2/20 | 310 | 2.8 |
| 13:40 | Ketamine 50 mg IV and clemastine 2 mg IV | — | — | — | — | — | — | — | — |
| 13:45 | Persistent bronchospasm | 110/50 | 85 | 0.80 | 40/4 | 6.3 | 6.2/20 | 310 | 2.5 |
| 14:00 | Methylprednisolone 80 mg IV. Phenylephrine infusion discontinued | 140/55 | 90 | 0.80 | 40/4 | 6.2 | 6.1/20 | 305 | 2.5 |
| 14:15 | Persistent bronchospasm | 150/55 | 85 | 0.80 | 40/4 | 6.4 | 6.1/20 | 305 | 2.6 |
| 14:30 | Additional methylprednisolone 420 mg IV | 135/55 | 85 | 0.80 | 40/4 | 6.6 | 3.6/20 | 180 | 2.6 |
| 14:45 | Persistent bronchospasm | 150/55 | 85 | 0.80 | 40/4 | 6.6 | 5.4/26 | 208 | 3.3 |
| 15:00 | Fentanyl 100 µg IV | 140/55 | 85 | 0.80 | 40/4 | 6.9 | 5.4/26 | 208 | 3.3 |
| 15:15 | Persistent bronchospasm | 130/60 | 80 | 0.80 | 40/4 | 6.9 | 5.7/26 | 219 | 3.3 |
| 15:30 | Insulin 6 IU administered | 120/55 | 85 | 0.80 | 40/4 | 7.4 | 5.7/26 | 219 | 3.3 |
| 15:45 | Persistent bronchospasm | 115/50 | 80 | 0.80 | 40/4 |  |  |  |  |
| 16:00 | Persistent bronchospasm | 115/50 | 80 | 0.80 | 40/4 | 7.2 | 6.4/26 | 246 | 3.3 |
| 16:15 | Persistent bronchospasm | 125/60 | 90 | 0.80 | 40/4 | 7.0 | 6.5/26 | 250 | 3.3 |
| 16:30 | Preparation for ECMO cannulation | 125/60 | 90 | 0.80 | 40/4 | 6.9 | 6.4/26 | 246 | 3.3 |
| 16:45 | Central venous access and urinary catheter inserted immediately before ECMO cannulation | 115/60 | 90 | 0.80 | 40/4 | 7.0 | 6.7/26 | 258 | 3.3 |
| 17:00 | VV-ECMO initiated; subsequent transfer to University Hospital | — | — | 0.80 | — | 7.0 | — | — | 3.4 |

**Supplementary Table 2B. Serial arterial blood gas measurements before and after VV-ECMO initiation**

| **Time** | **Relation to VV-ECMO** | **pH** | **PaCO₂ (kPa)** | **PaO₂ (kPa)** | **FiO₂** | **P/F ratio (mmHg)** | **Lactate (mmol/L)** |
| --- | --- | --- | --- | --- | --- | --- | --- |
| 13:16 | Before VV-ECMO | 7.18 | 8.7 | 14.6 | 0.60 | 183 | 2.5 |
| 14:03 | Before VV-ECMO | 7.12 | 9.8 | 15.8 | 0.80 | 148 | 2.8 |
| 15:07 | Before VV-ECMO | 6.97 | 14.4 | 13.5 | 0.80 | 127 | 2.4 |
| 15:44 | Before VV-ECMO | 7.01 | 12.6 | 14.6 | 0.80 | 137 | 3.1 |
| 17:18 | After VV-ECMO initiation | 7.12 | 7.9 | 16.9 | — | — | 5.5 |
| 18:56 | During VV-ECMO | 7.33 | 5.1 | 14.7 | — | — | 7.9 |
| 19:56 | During VV-ECMO | 7.37 | 4.5 | 17.4 | — | — | 9.4 |
| 21:30 | During VV-ECMO | 7.42 | 4.3 | 18.3 | — | — | 7.3 |

P/F ratios were calculated using the recorded PaO₂ and the corresponding ventilator FiO₂ after conversion of PaO₂ from kPa to mmHg.
